# MR Monitoring of Minimally Invasive Delivery of Mesenchymal Stem Cells into the Porcine Intervertebral Disc

**DOI:** 10.1371/journal.pone.0074658

**Published:** 2013-09-13

**Authors:** Monika Barczewska, Joanna Wojtkiewicz, Aleksandra Habich, Miroslaw Janowski, Zbigniew Adamiak, Piotr Holak, Hubert Matyjasik, Jeff W. M. Bulte, Wojciech Maksymowicz, Piotr Walczak

**Affiliations:** 1 Department of Neurology and Neurosurgery, Faculty of Medical Sciences, University of Warmia and Mazury, Olsztyn, Poland; 2 Department of Radiology, Faculty of Medical Sciences, University of Warmia and Mazury, Olsztyn, Poland; 3 Department of Surgery and Radiology, Faculty of Veterinary Medicine, University of Warmia and Mazury, Olsztyn, Poland; 4 Russell H. Morgan Department of Radiology and Radiological Science, Division of MR Research, the Johns Hopkins University School of Medicine, Baltimore, Maryland, United States of America; 5 Cellular Imaging Section and Vascular Biology Program, Institute for Cell Engineering, the Johns Hopkins University School of Medicine, Baltimore, Maryland, United States of America; 6 Department of Biomedical Engineering, the Johns Hopkins University School of Medicine, Baltimore, Maryland, United States of America; 7 Department of Chemical & Biomolecular Engineering, the Johns Hopkins University School of Medicine, Baltimore, Maryland, United States of America; 8 Department of Oncology, the Johns Hopkins University School of Medicine, Baltimore, Maryland, United States of America; 9 Department of NeuroRepair, Mossakowski Medical Research Centre, Polish Academy of Sciences, Warsaw, Poland; 10 Department of Neurosurgery, Mossakowski Medical Research Centre, Polish Academy of Sciences, Warsaw, Poland; University of Washington School of Medicine, United States of America

## Abstract

**Purpose:**

Bone marrow stem cell therapy is a new, attractive therapeutic approach for treatment of intervertebral disc (IVD) degeneration; however, leakage and backflow of transplanted cells into the structures surrounding the disc may lead to the formation of undesirable osteophytes. The purpose of this study was to develop a technique for minimally invasive and accurate delivery of stem cells.

**Methods:**

Porcine mesenchymal stem cells (MSCs) were labeled with superparamagnetic iron oxide nanoparticles (SPIO, Molday ION rhodamine) and first injected into the explanted swine lumbar IVD, followed by ex vivo 3T MRI. After having determined sufficient sensitivity, IVD degeneration was then induced in swine (n=3) by laser-evaporation. 3 x 10^6^ SPIO-labeled cells embedded within hydrogel were injected in 2 doses using a transcutaneous cannula and an epidural anesthesia catheter. T2-weighted MR images were obtained at 3T before and immediately after cell infusion. Two weeks after injection, histological examination was performed for detection of transplanted cells.

**Results:**

MSCs were efficiently labeled with Molday ION rhodamine. Cells could be readily detected in the injected vertebral tissue explants as distinct hypointensities with sufficient sensitivity. MR monitoring indicated that the MSCs were successfully delivered into the IVD *in* *vivo*, which was confirmed by iron-positive Prussian Blue staining of the tissue within the IVD.

**Conclusion:**

We have developed a technique for non-invasive monitoring of minimally invasive stem delivery into the IVD at 3T. By using a large animal model mimicking the anatomy of IVD in humans, the present results indicate that this procedure may be clinically feasible.

## Introduction

Back pain is one of the most frequent patient complaints, affecting millions of people worldwide and, in some cases, leading to long-term physical disability and a deteriorating quality of life. The pathophysiology of back pain is complex and not fully understood; however, a number of studies have implicated intervertebral disc (IVD) degeneration as a common cause of the disease [1-3]. Degenerative disc disease can occur at any age, and, due to its frequency, is most apparent in young adults, which can result in long-term absence from work and premature retirement. While surgical treatment and an appropriate rehabilitation plan may offer relief of acute symptoms, degenerative changes prevent recovery to the premorbid condition and usually lead to lifelong morbidity. Symptoms of the early IVD degeneration process are treated by minimally invasive NP vaporization using Percutaneous Laser Disc Decompression (PLDD) [4]; however, this treatment only relieves acute symptoms without any effect on the process of degeneration itself; thus, this procedure does not influence the natural course of the disease.

Thus, there is growing consensus that the efficacy of currently used therapeutic methods is inadequate, and new treatment strategies aimed at the full restoration of the IVD are being investigated. Regenerative medicine and tissue engineering approaches, such as cell-based therapies, are particularly promising in this regard. Mesenchymal stem cells (MSCs), with a demonstrated potential to differentiate toward multiple types of connective tissue cells [5], including those of the nucleus pulposus, represent an excellent option for the restorative treatment of degenerative disc disease. It has been shown that a robust differentiation of MSCs toward a chondrogenic/NP phenotype can be achieved *in vitro* with several methods, including the co-culture of MSCs with IVD explants [6], microencapsulation within alginate beads [7], or exposure to growth factors [8]. Moreover, transplantation of MSCs in animal models of degenerative IVD resulted in improvement of the IVD function [6,9,10].

However, it has been reported that transplanted cells can leak from the intervertebral space, which results in the formation of undesirable bone spurs (osteophytes), severely complicating the restorative processes [11]. In this context, the precision of cell delivery, and the ability to ensure that cells are deposited exclusively within the nucleus pulposus (without any leak to surrounding tissues) is of the utmost importance. Cellular MR imaging has recently emerged as the predominant imaging modality for cell tracking in animal models [12], as well as in patients [13-15]. Real-time monitoring of cell delivery by MRI might be instrumental for avoiding unwanted distribution of transplanted cells. Cells are made MR-visible by labeling with superparamagnetic iron oxide (SPIO) particles [16,17]. Despite the clear advantage of using MRI to track stem cells transplanted into the IVD, to our knowledge, as yet, the only published report refers to imaging of SPIO-labeled cells *in vitro* [18]. The population of patients targeted by PLDD may benefit specifically to the largest extent from cell-based repair due to the early stage of the disease where only moderate degeneration is present. In this report, we present a novel method of percutaneous, minimally invasive delivery of magnetically labeled MSCs into the IVD space, under precise MR monitoring of cell delivery.

## Materials and Methods

### Ethics Statement

All animal experiments described in this manuscript were conducted in accordance with the institutional guidelines for the care and use of laboratory animals and were approved by the University of Warmia and Mazury Ethics Committee and were performed in accordance with the ARRIVE guidelines.

### Anesthesia

All experimental procedures on animals were performed under the following anesthesia protocol: after premedication, intramuscular ketamine (30mg/kg), azaperon (strensil; 3 mg/kg), and subcutaneous atropine (0.05mg/kg) were administered. Pigs were anesthetized with propofol at 3mg/kg/hr, or as required based on the monitoring of respiratory rate and motion.

### Isolation and labeling of MSCs

The iliac crest bone from porcine donors (n=3, 30 kg) was punctured, and bone marrow aspirate was collected under sterile conditions. A phosphate-buffered saline (PBS)-diluted cell fraction of heparinized bone marrow was layered over a Ficoll density gradient (1.077 g/mL, GE Healthcare), followed by centrifugation at 400G at room temperature for 35 min. Nucleated cells were collected, diluted with two volumes of PBS, centrifuged twice at 100G for 10 min, and finally resuspended in culture medium. MSCs were maintained in a humidified atmosphere of 95% air and 5% CO_2_ at 37°C. Forty-eight hours prior to transplantation, MSCs cultured as a monolayer at 70% confluence were labeled with iron oxide nanoparticles (Molday ION/Rhodamine, Biopal; 25 µg Fe/ml). Molday ION was mixed with culture medium and added to cells for 48 hours under normal culture conditions [19]. For verification of their phenotype, paraformaldehyde-fixed cells were immunostained for MSC marker CD90 and for pan-leukocyte marker CD45 (Becton Dickenson). Fluorescent FITC-conjugated antibody (Invitrogen) was used for secondary detection.

### IVD vaporization procedure

**Figure 1:**
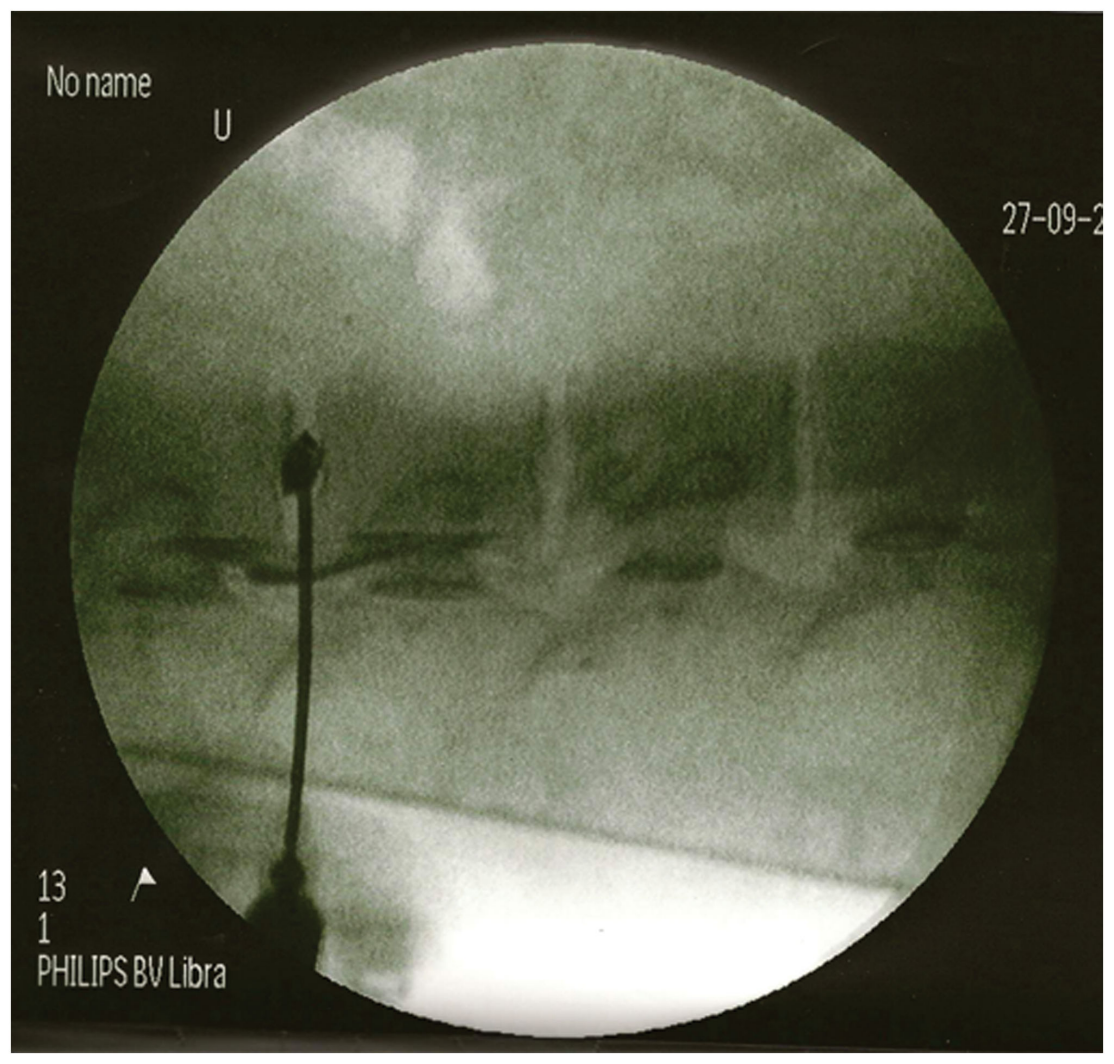
Targeting of the intervertebral disc under C-arm fluoroscopy. An epidural anesthesia needle was advanced under fluoroscopic guidance until reaching the center of the nucleus pulposus. Injection of Omnipaque contrast confirmed its proper localization.

Experiments were performed in juvenile female pigs (30 kg, n=3). Dehydration of the nucleus pulposus was induced by laser vaporization of the disc with an energy pulse of 1000 J over one minute (Dornier Medilas D MultiBeam, Germany). A laser fiberoptic probe was introduced into the intervertebral space using an epidural anesthesia needle under C-arm fluoroscopy (Siemens Medical Solutions) guidance (Figure 1). Proper positioning of the probe was confirmed by the injection of 0.5 ml Omnipaque contrast medium (Omnipaque; 300 mg Iohexolum/ml; Nycomed, Ireland). The animals were then positioned within the MRI scanner.

### Ex vivo MRI

For assessment of detection sensitivity at 3T, iron oxide-labeled MSCs (1x10^6^) were injected into a porcine verterbral column explant, and imaged using a T2 sequence (TE/TR=102/4500, AV=1, Res=560x560x3,000 µm).

### Cell transplantation and in vivo MRI

Immediately prior to transplantation, cells were washed with PBS, harvested by trypsinization, and suspended in HyStem hydrogel (Glycosan Biosystems) at a concentration of 1x10^6^/ml. Following NP evaporation by laser, the fiberoptic probe was replaced with a 26G plastic soft catheter. To avoid imaging artifacts, the metal needle was removed and the catheter was secured in place prior to scanning on a 3T MRI scanner (Magnetom TRIO, Siemens). A spinal surface coil was placed over the backs of the animals with cannulated discs. Pigs were positioned inside the scanner, with the plastic tubing extending outside the gantry to enable cell injection without the need to remove the animal from the scanner. A series of axial T2-w images (TE/TR=102/4500, AV=1, Res=560x560x3,000 µm) was acquired with the same coil geometry, without repositioning of the animal. Images were taken before and after the injection of 150µl/1.5x10^6^ cells, and again after the injection of a second dose of 150µl/1.5x10^6^ cells.

The change in signal intensity on pre- and post-injection MRI scans was investigated on T2-weighted images. ROIs that included the entire intervertebral disc were drawn for each of the four discs injected with SPIO-labeled cells, the three discs injected with non-labeled cells, and the five control, intact discs. Mean signal intensity per ROI was measured and subjected to statistical analysis. Since the surgical procedure and laser evaporation led to some hypointense changes, the intensity of pre-transplantation images was set at 100%, and the percent of signal change in post-transplantation images was analyzed and compared between experimental conditions.

After cell injection and imaging, the animal was removed from the scanner, the catheter was removed, and animals were allowed to recover from anesthesia.

### Tissue processing and histology

Terminally anesthetized pigs (propofol 5mg/kg) were transcardially perfused with 10 mM phosphate-buffered saline (PBS), followed by 4% paraformaldehyde (PFA) fixation at two weeks after transplantation. Intervertebral discs were removed and postfixed in 4% PFA overnight, cryopreserved in 30% sucrose, snap-frozen on powdered dry ice, and cryo-sectioned into 20 µm slices.

For the detection of intracellular SPIO in the MSCs residing in the IVD, the tissue was stained with Prussian blue. Briefly, fixed tissues were washed in distilled water and incubated for 30 min with 2% potassium ferrocyanide in 6% HCl, washed again, and counterstained with nuclear fast red. Slides were dehydrated and embedded with Histoclear mounting medium. Microscopy was performed using an Olympus BX51 fluorescence microscope equipped with a digital acquisition system.

### Statistical Analysis

PROC MIXED (SAS) was used to detect statistical significance, followed by the lowest means square (LMS) test for comparison between groups. The level of statistical significance was set at p=0.05.

## Results

### Culture of porcine MSCs and SPIO labeling

**Figure 2:**
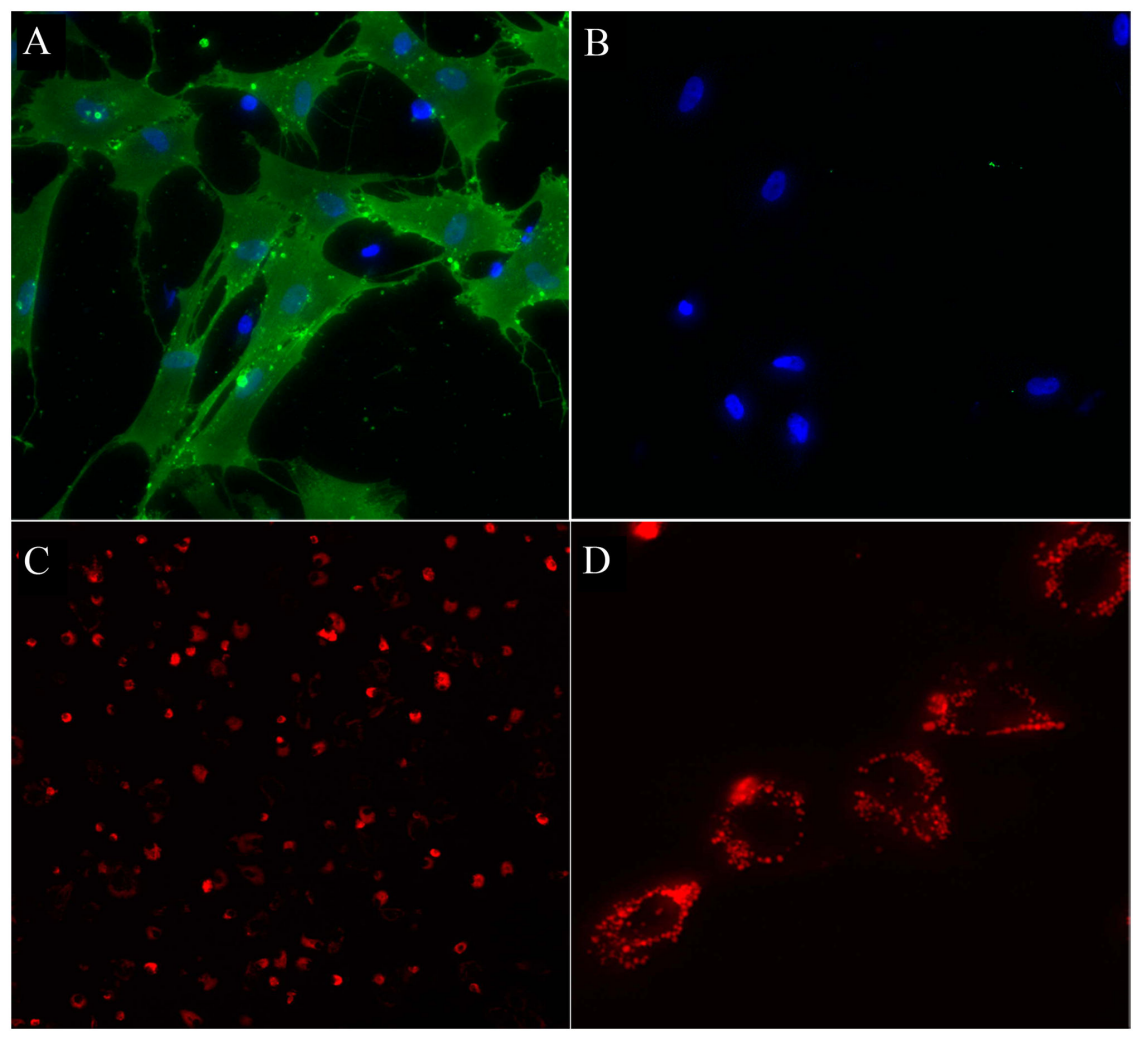
*In vitro* characterization of porcine MSCs. (A) Immunocytochemistry for CD90 antigen (*green*), Hoechst counterstain (*blue*). (B) Immunocytochemistry for CD45 (green), Hoechst counterstain (blue). (C) Fluorescent image of Molday ION-labeled MSCs (*red*); high power image shown in (D), confirming intracellular labeling.

Isolated porcine MSCs plated into a culture dish were spindle-shaped or multipolar and formed characteristic clusters indicative of cell proliferation. Immunocytochemistry anti CD90 revealed the expression of the mesenchymal stem cell marker in the majority (88.1 ± 2.5%) of cells (Figure 2A). Staining for the CD45 panleukocyte marker was negative (Figure 2B), thus further confirming the signature of the derived cells as mesenchymal. Magnetic labeling of MSCs with SPIO over a period of 48 hours was highly efficient and rendered nearly 100% of MSCs positive, as visualized by the red fluorescence of rhodamine (Figure 2C). SPIO particles were clustered within endosomes and distributed throughout the cytoplasm (Figure 2D). Notably, good labeling was achieved with this SPIO preparation without the need for the use of a transfection agent.

### Ex vivo MRI of MSCs injected into an IVD explant

**Figure 3:**
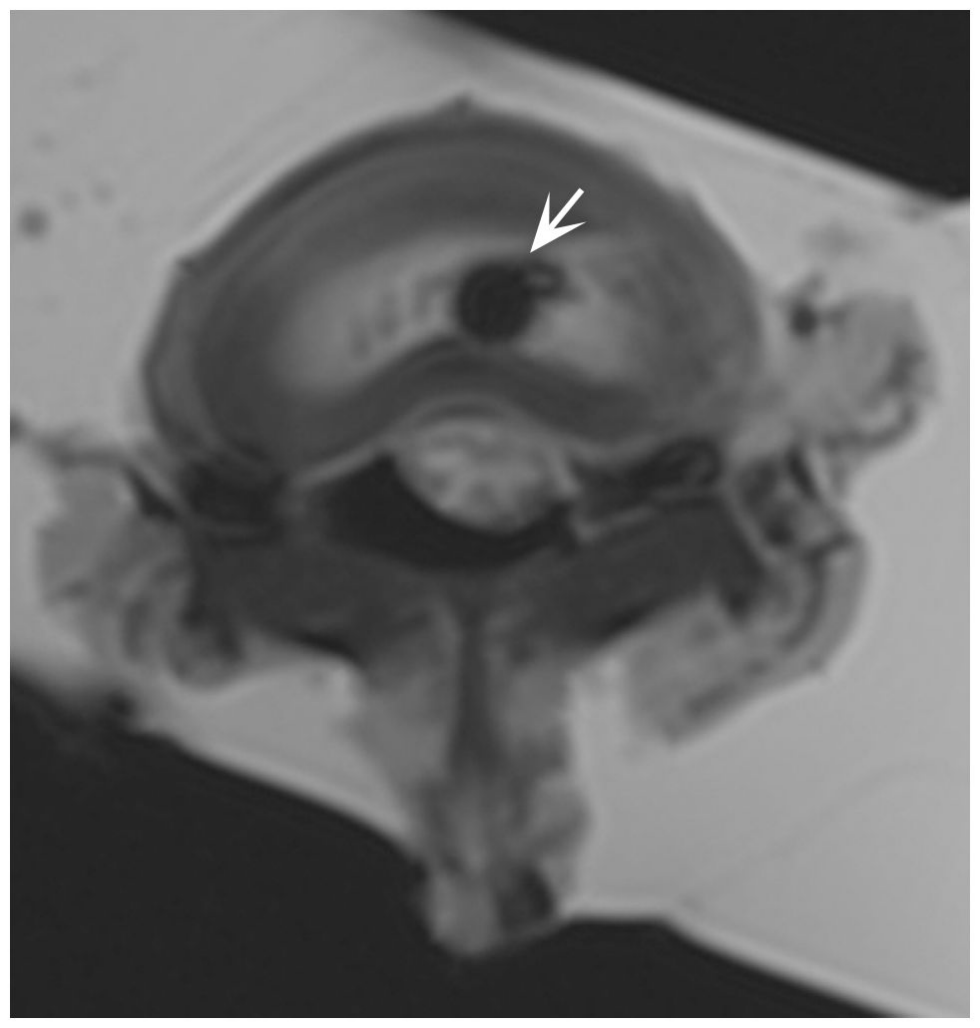
*Ex vivo* MR imaging of 1.0x10^6^ Molday ION-labeled MSCs injected in explanted spinal column. White arrow points to the hypointense region containing the cell deposit.

In order to establish whether the sensitivity of a clinical 3T scanner is sufficient for the detection of transplanted stem cells in the context of the intervertebral disc space, we dissected the lumbar region of the porcine spinal column from the surrounding muscles, and injected 1.0x10^6^ labeled MSCs centrally into the NP, using an 18G needle attached to an insulin syringe (BD, USA). The spinal column explant was then immersed in the saline and imaged on a 3T clinical scanner. The cells deposited into the center of the NP were clearly detectable on T2-weighted MRI scans as prominent hypointense regions (Figure 3B, arrow).

### Cell transplantation into the lesioned IVD under MR monitoring

**Figure 4:**
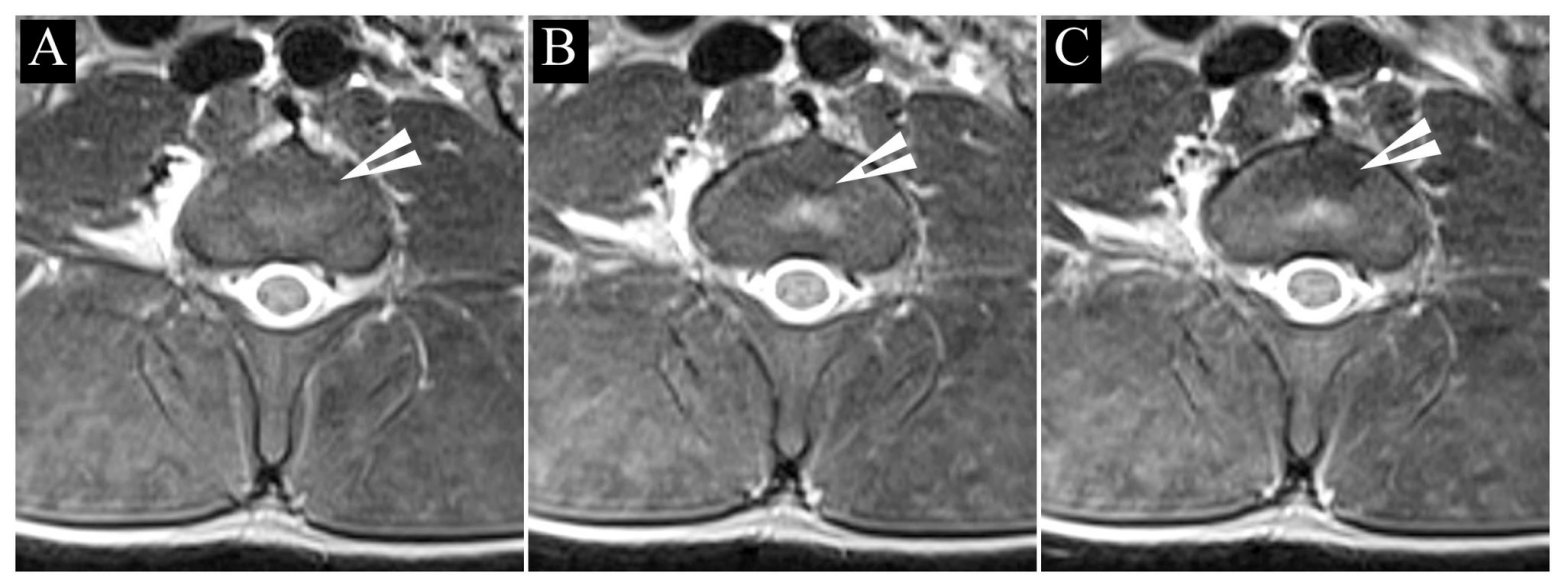
Serial *in vivo* axial MR imaging of (A) the lumbar region of the spine before cell injection, (B) after the first injection of 1.5x10^6^ Molday ION-labeled MSCs, and (C) after the second injection of 1.5x10^6^ labeled cells. White arrowheads points to the areas where the cells were deposited.

*In vivo* transcutaneous cell injection was initiated by the placement of an injection catheter within the IVD space under C-arm fluoroscopy guidance. Proper positioning of the probe was confirmed by the injection of Omnipaque contrast medium (Figure 1). The animals were then positioned within the MRI scanner. A T2-weighted scan prior to cell injection detected several hypointense regions around the vertebrae (Figure 4A, black arrowheads). These hypointensities are likely due to the presence of extravasated blood, as a consequence of the needle puncture. The injection of the first cell dose (1.5x10^6^ MSCs) resulted in a newly appearing hypointense region presenting within the intervertebral space (Figure 4B, white arrowhead). After injecting the second dose, the hypointense region significantly expanded as a result of the new influx of cells (Figure 4C, white arrowhead). For the three investigated groups, there was a statistically significant difference in the signal intensity reduction (p=0.004). In the SPIO-labeled cell group, the decrease in MRI signal was 10.6±5.1%. In the unlabeled cell group, there was only a 0.3±1.58% (p=0.004) change, and in control, uninjured discs, the change was 0.8 ±2.3% (p=0.002). There was no difference in signal intensity change between non-labeled cells and control discs (p=0.83), indicating that the procedure of cell injection does not influence the signal intensity, and that the significant decrease is selectively due to the injection of SPIO-labeled cells.

### Histology

**Figure 5:**
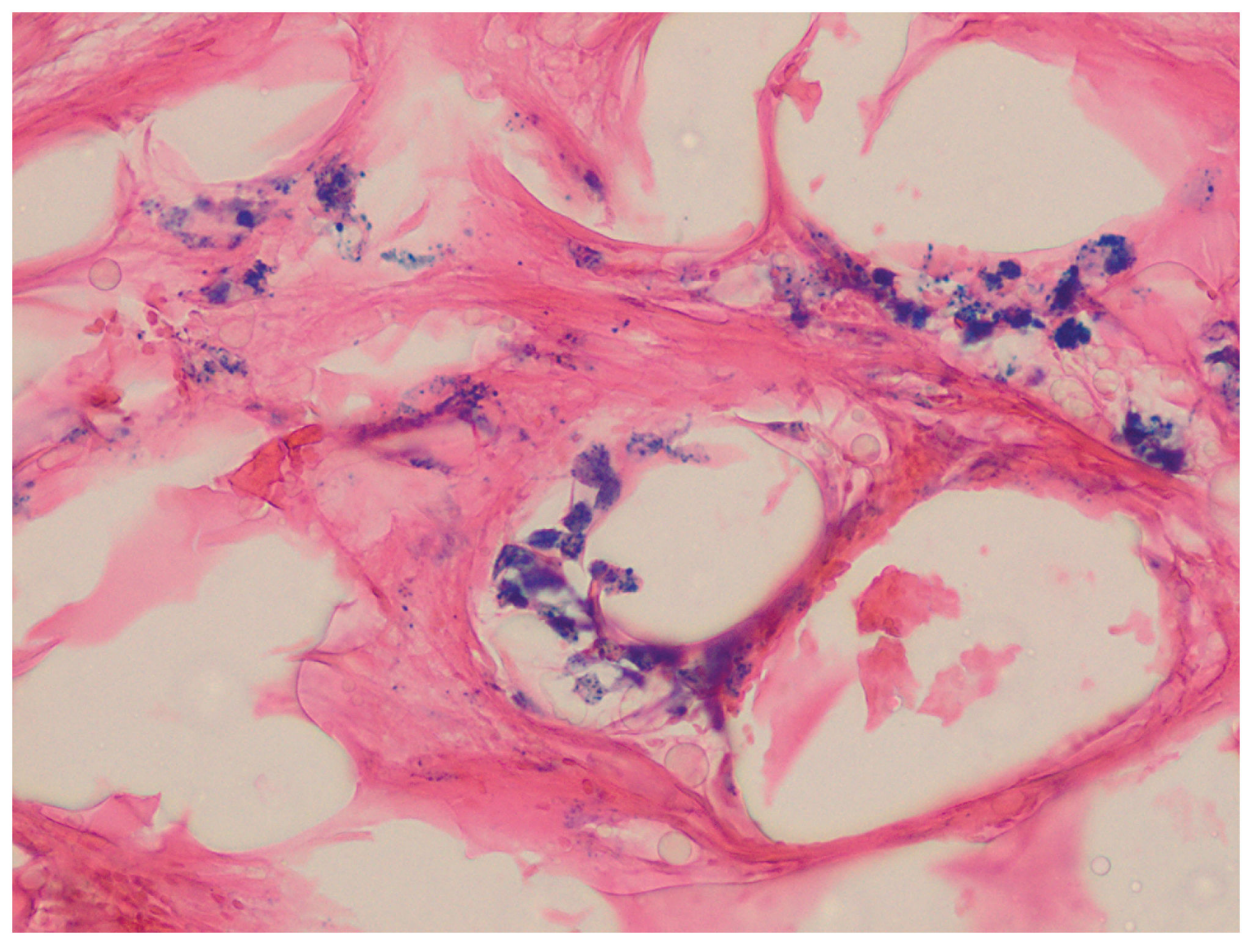
Prussian blue staining of disc tissue 2 weeks after injection of 3x10^6^ Molday ION-labeled MSCs. Iron positive blue precipitates are visible inside the transplanted MSCs.

To validate the imaging results and to confirm the presence of SPIO-labeled MSCs within the IVD, tissue was dissected, cryoprocessed, and stained for Prussian blue detection of iron. The presence of characteristic blue precipitates confirmed that there was a large amount of transplanted cells present throughout the disc tissue, that matched the anatomical location as seen on MRI (Figure 5).

## Discussion

The success of stem cell-based therapy for intervertebral disc degeneration (IVDD) depends on the efficient delivery and long-term retention of cells within damaged tissue, as well as on the appropriate differentiation of the cells and the production of an extracellular matrix. In recent years, significant progress has been made in the isolation and characterization of mesenchymal stem cells (MSCs), with a demonstrated potential to differentiate toward the cellular phenotypes of the nucleus pulposus. Transplantation of MSCs into animal models of IVDD has resulted in a functional benefit, as measured by the increased hydration and height of the treated intervertebral discs [20]. Moreover, initial pilot clinical trials with autologous MSCs were performed that also demonstrated some improvement [21]. These initially promising results should lead to the optimization of procedures for the safe and efficient delivery of these cells. In this study, we focused on establishing a technique for minimally invasive MSC injection into the nucleus pulposus, using accurate MRI visualization, in a clinically relevant porcine model.

Stem cell delivery to the degenerating disc is typically performed using percutaneous direct injection into the nucleus pulposus space, using an injection needle. This strategy has been tested in many models, including rats [22], rabbits [9], dogs [20], sheep [23], and pigs [24]. Mesenchymal stem cell transplantation has also been performed in patients who suffer from IVDD [21,25]. For these patients, autologous MSCs were either seeded onto a collagen sponge, with small fragments of the sponge grafted percutaneously [25], or injected as a suspension [21] into the area of the nucleus pulposus.

Our results showed that both PLDD and cell transplantation can be performed during the same procedure, thus facilitating the application of cell therapy. Our method of cell delivery was based on the introduction of MSCs, suspended in hydrogel, into the free space produced inside the disc by laser vaporization and suction. Pre-injection vaporization is important, as MSCs could then occupy the appropriate location without increased pressure during the injection and with the annulus fibrosus layers intact. This also diminishes the risk of cell leakage. Preventing leakage of implanted cells is especially important for the clinical translation of this method. Every effort must be directed toward avoiding the formation of osteophytes, which has been observed as a result of cell leak to surrounding tissues in experimental animal studies [11]. Labeling cells with iron oxide nanoparticles for MRI appears an excellent technique that enables precise, *in vivo* monitoring of the distribution of implanted cells.

However, the procedure of the introduction of the catheter into the IVD may result in some minor bleeding along the path to the IVD, which, in turn, may be mistaken for magnetically labeled cells. Thus, only interactive imaging (immediately before and after transplantation), is capable of providing a definitive answer about the location of transplanted cells. In fact, we have shown that the procedure of cell injection does not produce changes in the MR signal by itself, thus the decrease in signal is directly related to SPIO signal coming from cells, and could be used as marker of the accurate deposition of SPIO-labeled cells. As we have used a large animal model successfully, such a procedure should be readily translatable to the clinic.

There is an abundance of reports that indicate the feasibility of MRI tracking of MSCs in animal models of stroke [26] or spinal cord injury [27], and the first clinical MRI cell-tracking studies have now been performed [13], including those with MSCs [14]. At the time of this report, there have been no reports about the *in vivo* imaging of MSCs transplanted into the IVD. However, a study about the *ex vivo* MRI of MSCs injected into an explanted intervertebral space demonstrated the feasibility of this approach [18].

## Conclusions

Presented here is a novel method for real-time MRI monitoring of the percutaneous, minimally invasive delivery of magnetically labeled MSCs into the IVD space. We show that a clinical 3T scanner is suitable for the *in vivo* detection of labeled MSCs in the context of the IVD. Since all studies were performed in large animals and with the use of clinical equipment, this approach should be readily clinically translatable.
